# Cultural gems linked open data: Mapping culture and intangible heritage in European cities

**DOI:** 10.1016/j.dib.2023.109375

**Published:** 2023-07-05

**Authors:** Sergio Consoli, Valentina Alberti, Cinzia Cocco, Francesco Panella, Valentina Montalto

**Affiliations:** European Commission, Joint Research Centre (JRC), Via E. Fermi 2749, I-21027 Ispra (VA), Italy

**Keywords:** ICT and Society, Social applications of the Semantic Web, Linked Open Data for Cultural Heritage, Cultural domain ontologies, Semantic Web content creation, annotation, and extraction, Ontology mapping, merging, and alignment, RDF data processing

## Abstract

The recovery and resilience of the cultural and creative sectors after the COVID-19 pandemic is a current topic with priority for the European Commission. *Cultural gems* is a crowdsourced web platform managed by the Joint Research Centre of the European Commission aimed at creating community-led maps as well as a common repository for cultural and creative places across European cities and towns. More than 130,000 physical locations and online cultural activities in more than 300 European cities and towns are currently tracked by the application. The main objective of *Cultural gems* consists in raising a holistic vision of European culture, reinforcing a sense of belonging to a common European cultural space. This data article describes the ontology developed for *Cultural gems*, adopted to represent the domain of knowledge of the application by means of *FAIR* (*F*indable, *A*ccessible, *I*nteroperable, *R*eusable) principles and following the paradigms of Linked Open Data (LOD). We provide an overview of this dataset, and describe the ontology model, along with the services used to access and consume the data.

**Specifications Table**

**Table:** 

Subject	Arts and Humanities
Specific subject area	Ontology engineering, knowledge management, and linked open data technologies to model the European cultural heritage
Type of data	Text files Graphs OWL files RDF/XML files Turtle files Figures
How the data were acquired	Data was acquired by fetching *Cultural gems* (CG), a public application [1,2] designed and managed by the EC Joint Research Centre (DG JRC) [3]. It is a free and open-source web platform, crowdsourced, which maps cultural and creative venues in European cities. The *Cultural gems* app includes data on selected cultural venues from OpenStreetMap [4], and information provided by European cities, universities, public and private organizations, and other individuals that can share their data, to allow them to visualize the information on enriched city maps. Data resources have been also linked using **owl:sameAs** axioms to other public ontologies: DBpedia [5] and GeoNames [6]. Data is constantly expanding [7] and further alignments to other popular semantic datasets, freely accessible online and specialized in the cultural heritage domain, will follow. To design the linked open data model for *Cultural gems* we have directly mapped the main definitions from classes defined in the CG platform. These are primarily structured according to the OpenStreetMap classification [4] and the “concentric circles model of cultural industries” [8]. We also adopt ontology design patterns (ODPs) principles [9] to design the ontology. As such, we reuse ODPs already defined in other public ontologies as much as possible. We rely on the OPLa ontology [10] to identify these ODPs for an intuitive understanding of the overall class mapping of the ontology.
Data format	Raw Filtered
Description of data collection	Data were collected from the *Cultural gems* application and transformed to individuals of the produced ontology through an ETL (Extract-Transform-Load) nightly job. The code has been developed using the Python programming language. The resulting data ontology currently accounts for around 2.9M triples [7]. It is available in both RDF/XML and Turtle file formats. The reference namespace for the data is: https://culturalgems.jrc.ec.europa.eu/resource/. Instead, the reference namespace for the ontology definition is: https://culturalgems.jrc.ec.europa.eu/ontology/cultural-gems/, for a total of 67 classes. In our ontology engineering work, we have adopted commonly-accepted style rules for identifying and describing ontology objects. Specifically, data names inside the ontology have been expressed in lowercase, with dashes used in place of any potential space characters. Class names from ontology definitions, likewise, have been written in uppercase.
Data source location	European Commission, Joint Research Centre (JRC) Via E. Fermi 2749, I – 21027 Ispra, Varese Italy
Data accessibility	Source ontology definition and data files are available in RDF/XML and Turtle formats [7] within the Joint Research Centre Data Catalogue [11] at the following permanent location: https://data.jrc.ec.europa.eu/dataset/9ee32efe-af81-48e4-8ad6-a0db06802e03. These ontologies have been also released within the European Data portal [12], the official data repository for European data, at the following permanent location: https://data.europa.eu/data/datasets/9ee32efe-af81-48e4-8ad6-a0db06802e03. We enable the interested community to access and consume the produced data and ontology under Creative Commons Attribution 4.0 International (CC BY 4.0) license. Repository name: Joint Research Centre Data Catalogue
	Data identification number: http://data.europa.eu/89h/9ee32efe-af81-48e4-8ad6-a0db06802e03 Direct URL to data: https://data.jrc.ec.europa.eu/dataset/9ee32efe-af81-48e4-8ad6-a0db06802e03 Repository name: European Data portal Data identification number: http://data.europa.eu/88u/dataset/9ee32efe-af81-48e4-8ad6-a0db06802e03 Direct URL to data: https://data.europa.eu/data/datasets/9ee32efe-af81-48e4-8ad6-a0db06802e03 Data are freely available to the public and can be downloaded without any login details by selecting the appropriate URL in one of the repositories above. In particular, for the sake of clarity, we specify below the URL of each of the released data source: • Cultural gems ontology definition (RDF/OWL): https://jeodpp.jrc.ec.europa.eu/ftp/jrc-opendata/CC-COIN/cultural-gems/ontology-definition/cultural-gems.owl • Cultural gems ontology definition - no imports (RDF/OWL): https://jeodpp.jrc.ec.europa.eu/ftp/jrc-opendata/CC-COIN/cultural-gems/ontology-definition/cultural-gems-skeleton.owl • Cultural gems ontology individuals (RDF/OWL): https://jeodpp.jrc.ec.europa.eu/ftp/jrc-opendata/CC-COIN/cultural-gems/ontology-individuals/cultural-gems-resources.owl • Cultural gems ontology individuals (Turtle): https://jeodpp.jrc.ec.europa.eu/ftp/jrc-opendata/CC-COIN/cultural-gems/ontology-individuals/cultural-gems-resources.ttl We also enable direct access to the data and ontology by means of SPARQL queries on CELLAR [13], the Publications Office's common repository of metadata and content, by using its REST APIs SPARQL endpoint services. The REST web service to access the dedicated CELLAR SPARQL endpoint is https://publications.europa.eu/webapi/rdf/sparql

## Value of the Data

• Using information from individuals and organizations all around Europe, this data creates a unique crowdsourced archive of cultural and creative locations.
• The released ontology serves as a digital promotion tool for local government entities and anyone working within the arts and culture sector.
• The dataset may be beneficial for people concerned with research and development tools in the cultural heritage field.
• This data helps to interconnect practitioners and researchers exploiting the cultural heritage sector.
• Local authorities, research organizations, municipalities, and European institutions may be beneficial in the use of this data for the development of cultural information services and systems.
• An additional value is that the ontology is sharable, extensible, and easily reusable. Resources can be described jointly with other linked cultural datasets contributed by other communities, enabling interoperability at both syntactic and semantic levels.

## 1 Objective

The main objective of the released ontology consists of improving the data infrastructure of the European Commission's *Cultural gems* application. The objective is to allow proper management of cultural properties, events, stories, geo-locations (points, lines, polygons,...), intangible heritage, and the time dimension (one-time, recurrent). The ontology model further enhances information accessibility and interoperability. The goal is to support and encourage European organisations and individuals to interact with the application to build a unique database of cultural and creative locations. We want to support a shared awareness of European culture to reinforce the perception of one European cultural domain. For this purpose, the released ontology [7] exploits the large capabilities of Semantic Web technologies to connect with the various datasets and ontologies existing in the cultural domain [14]. We aim at enabling the adoption of several additional services and tools that we are currently building on top of CG to increase the final user experience.

## 2 Data Description

The primary classes employed in the platform, whose present taxonomy is roughly based on the “concentric circles model of cultural industries” [8], have been utilized for developing the released *Cultural gems* ontology. The ontology seeks to properly represent the cultural data of the application. For interoperability and simplicity, the classes have been mostly structured to reflect the OpenStreetMap categorization [4], useful to our mapping requirements.

Our designed ontology model reuses several properties and classes of ArCo [15], the knowledge graph of the Italian cultural heritage, utilizing *owl:imports* relationships. ArCo is used as the top-level taxonomy of the *Cultural gems* ontology. CG concepts are modelled as subclasses of ArCo in RDF/OWL standard format. In particular, we reuse the ArCo:*TangibleCulturalProperty* and:*IntangibleCulturalProperty* classes, defined as subclasses of:*CulturalProperty*. Furthermore, the:*TangibleCulturalProperty* concept is refined into the:*MovableCulturalProperty* and:*ImmovableCulturalProperty* classes. In addition, we further adopt the:*HistoricOrArtisticProperty*,:*ArchaeologicalProperty*, and:*MusicHeritage* cultural classes from ArCo. Exhibitions and events involving a cultural property have also been represented using the cultural events ontology of ArCo, which expands Cultural-ON [16].

**Fig. 1:**
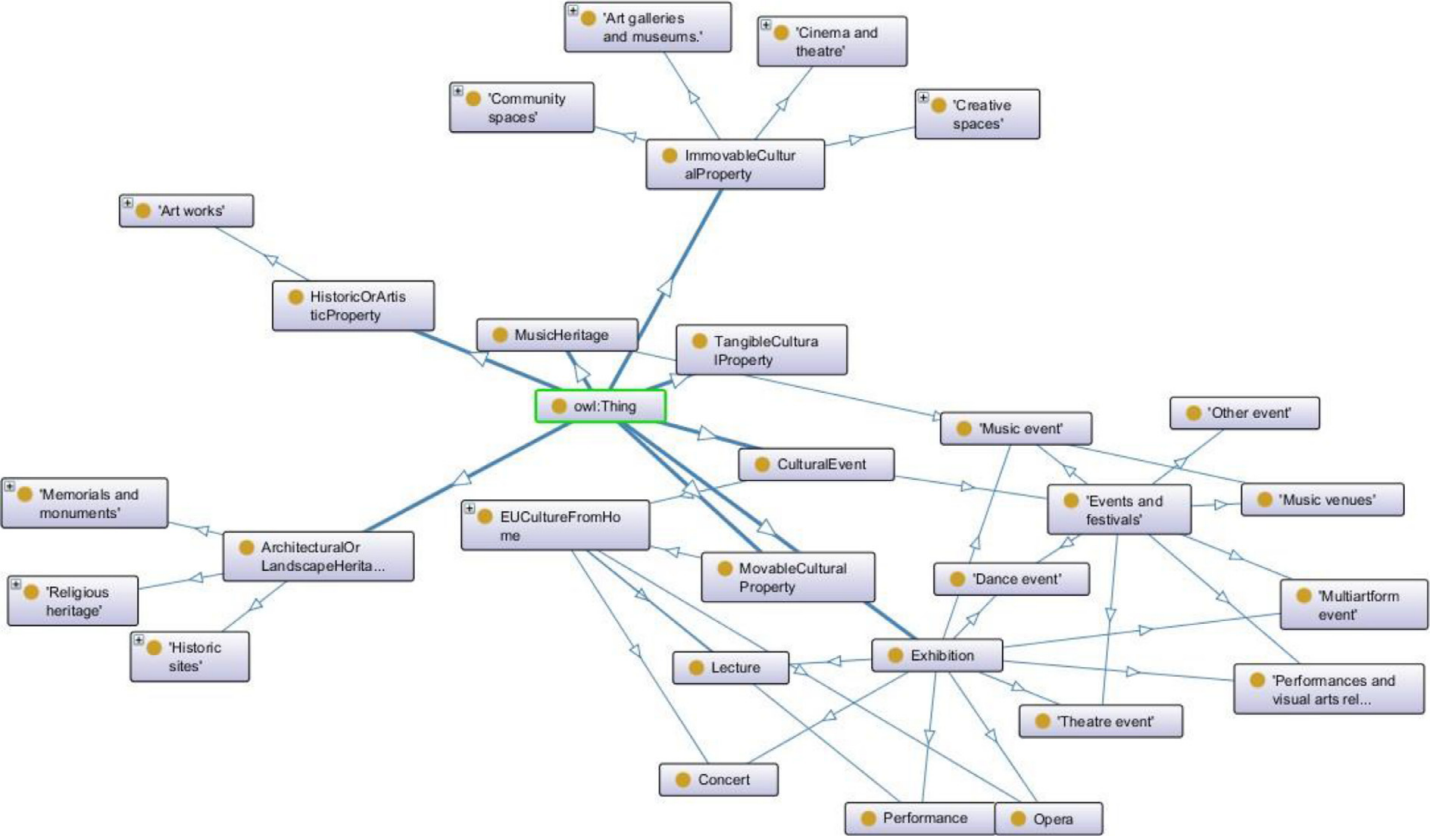
Main classes of the *Cultural gems* ontology.

Fig. 1 illustrates how the top classes of *Cultural gems* are connected to these ones. The primary class hierarchy of CG is described as follows.

The *EUCultureFromHome* class represents various cultural opportunities available online in EU cities. Travel and social restrictions caused by the Covid-19 pandemic recently limited our possibility to physically visit popular European cultural venues. As a reaction, several museums, theatres, local cultural organizations, libraries, and many more cultural venues organized various initiatives accessible online. This ontology class describes a set of performances and events organized to overcome physical distances.

The *Cinemas and theatres* class represents cultural venues such as cinemas, opera houses, theatres etc.

The *Art galleries and museums* class models cultural venues such as art galleries and museums, while the *Artworks* class describes works of art produced and exhibited outside of the conventional gallery environment.

The *Creative spaces* class depicts physical structures and elements at different scales that are deliberately designed to support creative work processes or to facilitate creativity.

The *Historic sites* class represents historic sites, that is official locations where pieces of political, military, cultural, or social history have been preserved due to their cultural heritage value. Historic sites, also referred to as heritage sites, are usually protected by law, and many have been recognized with the official national historic site status. A historic site may be any building, landscape, site or structure that is of local, regional, or national significance (meaning approximately 50 years or older).

The *Religious heritage* class defines spiritual and religious venues such as: churches, monasteries, crypts, shrines, sanctuaries, mosques, synagogues, temples, graveyards, sacred landscapes, sacred groves, and other landscape features, etc.

The *Memorials and monuments* class defines different types of historical attractions, while the *Events and festivals* class describes the several kinds of events and celebrations.

The *Community spaces* class describes the various types of social and community areas.

Finally, the *Music venues* class models any location utilized for a concert or performance of music, including practice and recording studios.

Geometric and spatial data have been modelled using ArCo's location module. As a result, a gem can be composed of more places, which are described by the *a-loc:LocationType* class in the ontology. In addition, a gem can be associated with a defined timespan. The time dimension is designed by adopting the *a-loc:TimeIndexedTypeLocation* class in the ontology, expanding the *TimeIndexedSituation* ODP.

The ontology definition namespace is https://culturalgems.jrc.ec.europa.eu/ontology/cultural-gems/, containing a total of 67 classes to date.

*Cultural gems* data populating the application are modelled as individuals assigned to a class of the designed ontology definition. The resulting data ontology accounts for around 2.9M triples to date. It is publically available in both Terse RDF Triple Language (Turtle) and RDF/XML formats [7]. The data ontology namespace is https://culturalgems.jrc.ec.europa.eu/resource/.

Data resources are currently linked, whenever is appropriate, tp DBpedia [5] and GeoNames [6] with **owl:sameAs** relationships. The ontology is continuously expanding with additional alignments to other public cultural ontologies, such as Europeana and ArCo data, which will come in the future.

Ontology data names are expressed in lowercase, and eventual space characters have been replaced by dashes, as per common style guidelines for naming and labeling ontologies [9]. Differently, ontology definition class names are expressed in uppercase, following common style guidelines used for naming and labeling ontology definitions.

Both the definition and data ontologies are available [7] within the Joint Research Centre Data Catalogue [11] at the following permanent location: https://data.jrc.ec.europa.eu/dataset/9ee32efe-af81-48e4-8ad6-a0db06802e03, and within the European Data portal [12], the official data repository for European data, at the following permanent location: https://data.europa.eu/data/datasets/9ee32efe-af81-48e4-8ad6-a0db06802e03.

## 3 Experimental Design, Materials and Methods

Ontologies need to be appropriately created, maintained, adjusted, and expanded, as required. For these purposes, the developed ontology has been managed by adopting the *Protègè* ontology editor. *Protègè* provides a fully integrated interface allowing to add, for example, new classes, or to update already existing ones.

Ontology design patterns (ODPs) principles [9] have been used in the modelled ontology. Whenever appropriate, existing ODPs from popular ontologies have been reused using OPLa ontology [10]. For example, we have linked to classes and properties from the OntoPia Public Administration vocabulary and from Cultural-ON, a popular ontology modelling cultural locations and events [16]. The adoption of OPLa for annotating the reuse of other ontologies supports the overall class identification and mapping of the overall ontology.

*Cultural gems* data in the application populates the CG ontology via an ETL (Extract-Transform-Load) nightly job developed in Python. Data are modelled as individuals of the ontology definition. These individuals are linked to data of other external ontologies by means of LIMES [17], a popular link discovery tool for LOD.

The ontology definition is stored in the RDF named-graph: https://culturalgems.jrc.ec.europa.eu/ontology/cultural-gems/, while ontology resources are stored in the RDF named-graph: https://culturalgems.jrc.ec.europa.eu/resource/.

For programmers, both ontology definition and data are interrogable via SPARQL queries on a dedicated Virtuoso triplestore. For this purpose, we are leveraging CELLAR [13], the official triplestore of the Publications Office of the European Union. Queries are possible via the dedicated REST APIs available to the Virtuoso endpoint of CELLAR. Queries can be made by editing the text area available in the interface for the SPARQL query language. SPARQL is the standard language reference and a W3C recommendation for querying RDF data. The REST APIs to the CELLAR triplestore [13] need a specific query formatted in the SPARQL language as input, giving as output the query result in *text/html, text/rdf +n3, application/xml, application/json*, or *application/rdf+xml*. The specific format is defined by the user as desired.

CONSTRUCT, ASK, DESCRIBE, SELECT queries can be performed to access the CG ontology. A CONSTRUCT query returns an RDF source constructed by substituting variables. An ASK query returns true or false indicating whether a query pattern matches or not. A SELECT query returns the variables bound in a query pattern match.

For example, we are given the Ancienne Belgique - AB gem, a popular concert hall for contemporary music located in Brussels, Belgium, and the related resource in the data ontology corresponds to https://culturalgems.jrc.ec.europa.eu/resource/cultural-gems/139885.

To obtain all the RDF information related to this resource from the CELLAR endpoint, the corresponding SPARQL query is:

SELECT * WHERE < https://culturalgems.jrc.ec.europa.eu/resource/cultural-gems/139885 *> ?p ?o*

In simple words, it translates into the selection of all the data triples having properties ?p and objects ?o, matching with the resource “Ancienne Belgique - AB” specified as subject.

The ontology can also be exploited by using the *Live OWL Documentation Environment (LODE)* [18], a service that visualizes classes, object properties, data properties, named individuals, annotation properties, general axioms and namespace declarations from an RDF/OWL ontology, in a human-readable HTML page. This tool enables user-oriented navigation and provides an intuitive description of elements of the ontology.

**Fig. 2:**
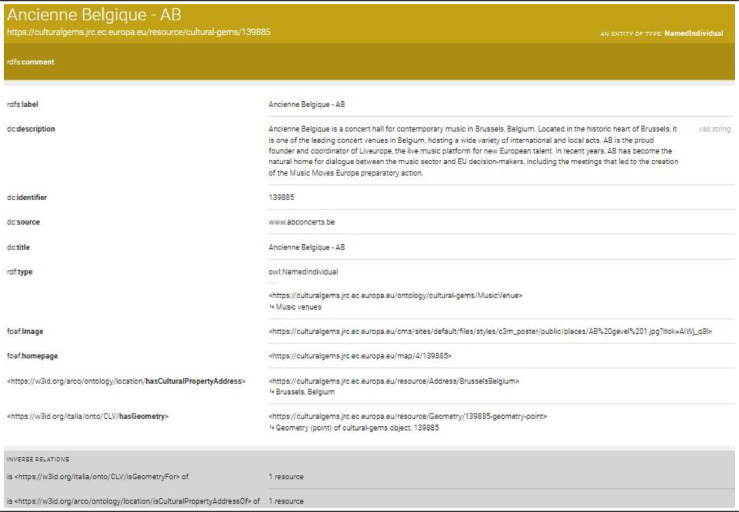
IRI dereferentiation of the Ancienne Belgique - AB gem by means of LodView.

We also enable IRI dereferentiation by using LodView [19], a Java web application compliant with W3C standards for content negotiation. LodView improves the end user's experience by providing an HTML page describing the ontology data. Besides its intuitive interface, LodView adds interesting features to the content negotiation, such as the possibility to download the selected resource in different formats (*xml, ntriples, turtle, ld+json*). For example, the Ancienne Belgique - AB gem can be visualized via LodView at Ancienne Belgique - AB lodview (Fig. 2). With this representation, users can explore and navigate the resource, noting e.g. that this gem is a subclass of the Music Venue class, and has associated a main source homepage. It is also possible to download the raw information of the resource in various formats, e.g. json or csv, among others.

**Fig. 3:**
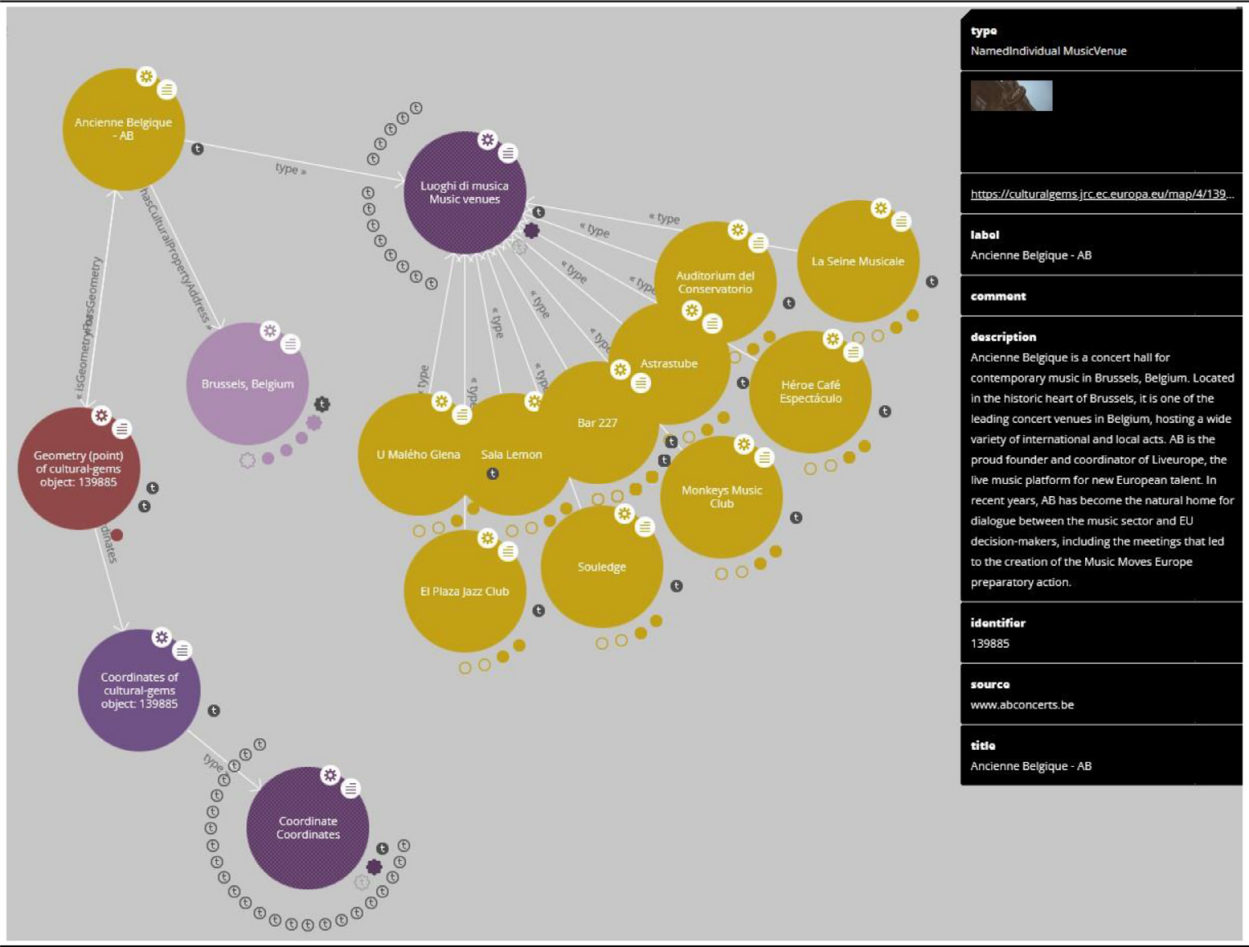
Graph visualization of the Ancienne Belgique - AB gem by means of LodLive.

We further integrate LodLive [20] to facilitate the navigation of the data in the ontology. LodLive enables the visualization of the resources of an ontology as an efficient graph. The user can, for example, examine the structure of the RDF/OWL data and interactively explore the relationships of a particular ontology individual. LodLive also enables the association of geo-coordinates and images to the data resources and the computation of inverse and **owl:sameAs** links. As an example, Fig. 3 shows how the Ancienne Belgique - AB gem is interconnected with the other ontology individuals by means of the LodLive graph visualization.

## Ethics Statements

The present work did not involve human subjects, animals or information from social media platforms.

## CRediT authorship contribution statement

**Sergio Consoli:** Conceptualization, Data curation, Methodology, Software, Supervision, Writing – original draft. **Valentina Alberti:** Investigation, Visualization, Supervision, Writing – review & editing. **Cinzia Cocco:** Software, Investigation, Validation, Writing – review & editing. **Francesco Panella:** Data curation, Investigation, Supervision, Writing – review & editing. **Valentina Montalto:** Investigation.

## Declaration of Competing Interest

The authors declare that they have no known competing financial interests or personal relationships that could have appeared to influence the work reported in this paper.

## Data Availability

Cultural gems ontology: Mapping culture and intangible heritage in European cities (Original data) (European Data Portal).
